# Trans-Cinnamaldehyde as an Environmentally Low-Impact Phytosanitary: Evaluation of Its Toxicity Toward Aquatic and Terrestrial Non-Target Species

**DOI:** 10.3390/jox16030086

**Published:** 2026-05-16

**Authors:** Natalia Ferrando, Elisa Langa, Laura Botello-Morte, Pedro Rodríguez-López, Diego Ballestero, María Rosa Pino-Otín

**Affiliations:** 1 Facultad de Ciencias de la Salud, Universidad San Jorge Autovía Mudéjar, km. 299, 50830 Villanueva de Gállego, Spain; nferrando@unizar.es (N.F.); lbotello@usj.es (L.B.-M.); prodriguezl@usj.es (P.R.-L.); dballestero@usj.es (D.B.); rpino@usj.es (M.R.P.-O.); 2 Instituto Agroalimentario de Aragón-IA2, CITA-Universidad de Zaragoza, C. de Miguel Servet, 177, 50013 Zaragoza, Spain

**Keywords:** *Trans*-cinnamaldehyde, ecotoxicity, *Daphnia magna*, *Aliivibrio fischeri*, *Eisenia fetida*, *Allium cepa*, water microbiota, soil microbiota, Biolog Ecoplate

## Abstract

*Trans*-cinnamaldehyde (CIN), the main component of cinnamon essential oil, is a promising sustainable alternative to synthetic pesticides. Despite its use, ecotoxicological data on non-target species remain fragmented. This study systematically evaluates CIN’s acute toxicity across multiple trophic levels to characterize the biological sensitivity and environmental response of key organisms. Aquatic assays measured bioluminescence inhibition in *Aliivibrio fischeri* and immobilization in *Daphnia magna*. Terrestrial evaluations included lethality tests on *Eisenia fetida* and root elongation in *Allium cepa*. Additionally, the impact on soil and river microbial communities was analyzed via Biolog EcoPlates™. Significant dose–response relationships were observed across all bioindicators (*p* < 0.0001). *A. fischeri* was the most sensitive species (EC_50_ = 1.428 mg·L^−1^), followed by *D. magna* (EC_50_ = 4.533 mg·L^−1^). In terrestrial models, *A. cepa* (EC_50_ = 11.644 mg·L^−1^) exhibited higher sensitivity than *E. fetida* (LC_50_ = 412.519 mg·kg^−1^). Microbial metabolic activity showed dose-dependent inhibition, particularly affecting carbohydrate and polymer degradation at high concentrations. These findings define the first ecotoxicological benchmarks for CIN, establishing EC_10_ and EC_50_ values under standardized conditions. These data provide the necessary toxicological constraints to ensure environmental safety in future field-scale applications of this natural compound.

## 1. Introduction

*Trans*-cinnamaldehyde (CIN), an aromatic aldehyde naturally occurring as the major constituent of cinnamon bark and leaf oils, is used as a flavoring agent, fragrance ingredient, antimicrobial compound in food, cosmetic and industrial applications [1]. Moreover, CIN has demonstrated multifaceted utility as fungicide, larvicide, insecticide and antibiotic adjuvant highlighting its potential for integrated pest management strategies [2,3,4,5,6,7].

In recent years, there has been a notable shift towards the utilization of less toxic pesticides to mitigate the potential risks associated with environmental contamination, encompassing soil and water, while concurrently reducing hazards to human health and preserving the integrity of the soil microbiome, a pivotal component for ecosystem functionality [1,8]. Consequently, CIN has also been incorporated as the main active ingredient in commercial biopesticides based on cinnamon extract, reflecting a broader shift towards plant derived pesticides that are perceived as safer alternatives to conventional synthetic ones. For example, commercial formulations using cinnamon extract and pure CIN are being developed and applied in agricultural systems for the control of plant pathogens and pests, as well as in other sectors for microbial control, including corrosion related biofilms, leading to the formulation of commercial products with high concentrations of this compound [1,9].

However, stringent regulatory measures, particularly in regions such as the European Union (EU), have impeded the widespread commercialization of biopesticides, resulting in a significant disparity in availability between areas such as the EU and the United States of America (USA) [1,10]. Consequently, only 60 to 80 biopesticides are registered in the EU, in contrast to 200 to 400 in the USA [1,10]. Additionally, microbial biopesticides make up 63% of the biopesticides available in the global market [1]. Therefore, the expansion of biopesticide use remains constrained, with regulatory hurdles hindering the introduction of new products into the market [1].

This disparity in regulatory frameworks underscores the need for comprehensive evaluations of the efficacy and environmental impact of biopesticides to facilitate their broader adoption. Moreover, the resurgence of interest in organic agriculture, predicated on biological and cultural pest management practices, further emphasizes the imperative for sustainable pest control solutions [11,12,13].

Nevertheless, despite the generally favorable hazard profiles of biopesticides (Category III and IV) by the Environmental Protection Agency (EPA) of the USA [11,14], their toxicological implications and environmental persistence necessitate thorough investigation [11,15,16,17]. Additionally, the application of biopesticides at higher concentrations than naturally occurring compounds raises concerns regarding their ecological implications, underscoring the importance of understanding their mobility and fate in the environment [11,18,19,20,21].

In contrast to the growing body of data on the pesticidal and antimicrobial efficacy of CIN, information on its ecotoxicity to non-target terrestrial and aquatic organisms remains fragmented and comparatively scarce. Most ecotoxicological research using standard model species such as *Aliivibrio fischeri*, *Daphnia magna* or *Eisenia fetida* has focused on pharmaceuticals, personal care products and conventional pesticides, and has generated extensive datasets for these contaminants, but not for plant derived biopesticides such as CIN [1]. Consequently, generic assumptions about the safety of natural compounds should be avoided until systematic, multi trophic ecotoxicological evidence is gathered. Furthermore, there is a notable lack of integrated studies that simultaneously address different environmental compartments (freshwater vs. soil), taxonomic groups (microorganisms, invertebrates, plants) and levels of biological organization (from microbial communities to individual level endpoints such as survival, growth or genotoxicity) for this compound.

The central hypothesis of this study is that natural substances possess a more favorable ecotoxicological profile than conventional synthetic pesticides. In light of the considerations previously exposed, the primary objective of this study was to conduct a comprehensive ecotoxicological assessment of CIN across multiple trophic levels. By utilizing a battery of standardized bioassays, we evaluated its acute toxicity in aquatic organisms (*A. fischeri* and *D. magna*) and terrestrial species (*E. fetida* and *Allium cepa*). Furthermore, the research aimed to characterize the impact of CIN on the metabolic activity of microbial communities in both river water and soil environments, for a more realistic interpretation from an environmental point of view.

## 2. Materials and Methods

### 2.1. Reagents

*Trans*-cinnamaldehyde (CIN) (CAS number 104-55-2) was purchased from Sigma-Aldrich (Merck, St. Louis, MO, USA) with a purity higher than 95.0%.

### 2.2. Average Well Color Development (AWCD) Tests of Soil and Water Microorganisms

#### 2.2.1. Water Samples

Water sampling procedures adhered to standard methodologies [22], Figure S1. The water temperature was recorded at 17 °C, and the pH level was neutral. These measurements were performed in situ (Gállego River in Zaragoza, Spain, 41041′57″ N, 0049′1″ W, in June 2022). Detailed physico-chemical characteristics were previously presented in the work conducted by Ferrando et al. [23].

1 L of river water was filtered through a 70 μm nylon filter (Becton Dickinson, Madrid, Spain) to remove debris, and the filtered sample was stored at 4 °C for subsequent Biolog Ecoplate^TM^ (Tiselab S.L., Barcelona, Spain) experiments.

#### 2.2.2. Soil Samples

The soil preparation procedure was conducted in accordance with previously published methods [23,24,25,26], Figure S1. The sample was collected in June 2022 from a contaminant-free crop field at CITA in Zaragoza, NE Spain. The physico-chemical characteristics of the soil were previously published [23].

Initially, the sample was sieved to remove any contaminants larger than 2 mm and subsequently stored in sterile plastic bags in darkness. Then, in a laminar flow biological safety hood (Model MSC Advantage 1.2) to ensure sterility, 95 mL of distilled, filtered water (Sterifix^®^ 0.22 µm, Sartorius, Göttingen, Germany) was added to 10 g of soil. After stirring this mixture for 30 min, we allowed it to stand for 1 h, and then 6 Falcon tubes were each filled with 10 mL of the supernatant. The resulting 60 mL of leachate was then filtered using a 70 μm nylon filter to remove contaminants. The filtered sample was stored at 4 °C until the Biolog Ecoplate^TM^ assays.

#### 2.2.3. Biolog Ecoplate^TM^ Preparation

The microbial level physiological profile, which is the metabolic capacity for degrading various carbon sources after xenobiotic exposure (CIN in our study), was assessed using Biolog EcoPlates^TM^. These plates contained 3 replicates of 31 key organic substrates crucial for microbial metabolism, along with a water control [27]. This method evaluates the comprehensive impact of potentially harmful compounds on the metabolic functions of microbial communities [26,28,29].

Each well of the Biolog EcoPlates^TM^ was filled with 75 μL of processed water or soil leachate from the previous sections and 75 μL of CIN aqueous solutions at several concentrations (0.1, 10, 100, and 1000 mg·L^−1^), with 3 replicates for each concentration. The final pH of the dilutions was 6.5 ± 0.5. The plates were incubated (J.P. Selecta) under sterile conditions for 7 d at 25 °C. Optical density (OD) of each well was measured at 590 nm using a BioTek Synergy H1 microplate reader (BIO-TEK, Dallas, TX, USA) and Gen5TM (version 2.0) data analysis software, initially at t = 0 h and then every 24 h. These OD values allowed us to calculate the Average Well Color Development (AWCD) for the whole plate according to Equation (1) [25,30]:

AWCD = ∑(AbsW − AbsC)/31(1)

where AbsW is the OD of each well containing the carbon source and AbsC is the OD of the control well lacking the carbon source.

For a more specific analysis, the 31 substrates were grouped into 5 functional classes: amines/amides, amino acids, carboxylic and acetic acids, polymers, and carbohydrates [31]. Then, Equation (1) was employed to calculate the AWCD of each metabolic group [25].

To analyze if statistical signification was reached when comparing the evolution of absorbance over time, repeated measures ANOVA test was chosen, with post hoc comparisons by Sidak. ANOVA was carried out separately for each concentration and in a comparative way with the control as a factor. SPSS (Version 29.0.2.0(20), IBM SPSS Statistics) software was used with a threshold value of *p* = 0.01 to accept or reject null hypothesis.

### 2.3. Aliivibrio fischeri Bioluminiscence Assay

The objective of this experiment was to evaluate the toxicity of CIN by assessing the inhibition of natural bioluminescence produced by the marine bacterium *Aliivibrio fischeri* [32]. Lyophilized bacteria, purchased from Macharey-Nagel (ref. 945 006), were stored at −18 °C. The experimental protocol followed UNE-EN ISO: 11348-3:2007.165 [32], Figure S2. The lyophilized *A. fischeri* bacteria were rehydrated using the reactivation solution provided by the supplier and kept at 4 °C for 5 min.

A CIN stock solution of 4000 mg·L^−1^ was prepared by dissolving it in an aqueous solution of 20 g/L NaCl. To ensure proper oxygenation, the sample was vigorously stirred. In addition, the pH was monitored to remain within the specified range (6 to 8.5). Additionally, approximately 10 mL of culture medium (purchased from Macharey-Nagel, ref. 945 006) was added to the lyophilized vial to create the bacterial solution. Four replicates were performed for each sample dilution, and they were maintained at a temperature of 15 ± 1 °C.

*A. fischeri* solution bioluminescence was measured after 10 min of resting. Then, 0.5 mL of bacterial solution was exposed to each concentration (0.2, 2, 20, 200, and 2000 mg·L^−1^) of CIN dilutions and, after 30 min of exposure, the bioluminescence was measured again to calculate bioluminescence inhibition.

Using the XLSTAT software (version 2025.1.3 (1431), Addinsoft, Lumivero, Paris, France 2025), the EC_50_ and EC_10_ values, representing the effective concentrations of CIN that inhibited bioluminescence by 50% and 10%, respectively, along with their associated Confidence Intervals (CI), were calculated based on the dose–response curves for *A. fischeri*.

### 2.4. Daphnia magna Immobilization Assay

Tests were conducted on *Daphnia magna* (water flea) sourced from Vidrafoc (Barcelona, Spain) with reference DM121219, following standardized procedures [33,34] and the operational recommendations of the Daphtoxkit FTM Magna (1996), Figure S2. The planktonic crustaceans were stored at 5 °C prior to use, and their eggs were incubated for 72 h at 21 ± 1 °C in a TOXKIT model CH-0120D-AC/DC incubator (provided by ECOTEST, Valencia, Spain) with an illumination of 6000 lx. Crustaceans were fed with spirulina, provided in the Daphtokit, 2 h before exposure to CIN.

CIN was tested at 4 concentrations (0.1, 1, 10, and 100 mg·L^−1^) in sterile freshwater [34], which served as negative control. The pH of the water was adjusted to a range of 7.25 ± 0.25 using a 0.1 M NaOH solution. Each concentration was assessed with 5 replicates, each containing 5 organisms. Daphnia was exposed to the respective concentrations for 24 h under complete darkness at 21 ± 1 °C. Immobility was determined by gently agitating the organisms for 15 s; those unable to swim were considered immobile.

The effective concentration values of CIN resulting in 50% and 10% immobilization (inactive neonates) of *D. magna*, that is EC_50_ and EC_10_, and their associated CI were derived from dose–response curves using the XLSTAT software (version 2025.1.3 (1431), Addinsoft, Lumivero, Paris, France 2025).

### 2.5. Eisenia fetida Lethality Assay

Adult earthworms of *Eisenia fetida* were procured from Todo Verde (Ourense, Spain) and maintained in sphagnum peat substrate obtained from Spanish Flowers Company (Zaragoza, Spain), following the provider’s recommendations for optimal development. The earthworms were kept at controlled conditions (18–25 °C, pH 7.5–8, and humidity 80–85%) for a period of 14 d prior to testing. Standardized procedures [35] were followed during the experiments, Figure S2. Earthworms aged at least 2 months, exhibiting clitella, and weighing between 300 and 600 mg each were selected for the tests [24,36].

The experiments were conducted in 750 cm^3^ plastic jars equipped with lids to prevent the escape of animals and to maintain proper humidity levels. Holes were pierced in the jar lids to facilitate ventilation and oxygen supply. Each jar was filled with 750 g (wet weight) of standardized soil substrate composed of industrial fine sand, sphagnum peat, and kaolin clay in a ratio of 7:1:2, respectively [35]. Kaolin clay and sand were sourced from Imerys Ceramics (Onda, Spain), while sphagnum peat was obtained from Verdecora vivarium (Zaragoza, Spain). The moisture content of the mixture was adjusted to 35–45% of the dry weight of the soil by adding deionized water and thoroughly mixing it after weighing the sample and drying it to a constant mass at 105 °C for 24 h.

Ten earthworms were introduced into the jars with CIN at various concentrations (0.1, 1, 10, 100, 1000 mg·kg^−1^) for a duration of 14 d at 20 ± 2 °C, 80–85% relative humidity and 400–800 lx of light. Each concentration was tested 3 times, and negative controls without CIN were also included.

The lethal concentration values of CIN (LC_50_ and LC_10_) and their associated CI were determined from the dose–response curves for *E. fetida* tests using the XLSTAT software (version 2025.1.3 (1431), Addinsoft, Lumivero, Paris, France 2025).

### 2.6. Allium cepa Root Growth Assay

Bulbs of *Allium cepa* (Stuttgarter Riesen variety, 14/21, obtained from Fitoagrícola Company, Castellón de la Plana, Spain) were stored in a dry environment (10–20 °C) in darkness until utilized. Prior to testing, the young bulbs were peeled to preserve the root ring.

Acute toxicity experiments involving *A. cepa* were conducted following specific standardized procedures [37], Figure S2, which assessed root elongation after 72 h of exposure to CIN. Mineral water from Aguas de San Martín de Veri S.A., Bisaurri, Spain, (https://www.veri.es/es/el-producto (accessed on 1 May 2026)) utilized as the growth medium for the bulbs, was placed in 15 mL tubes. This water was chosen because it contained sufficient levels of Ca^2+^ and Mg^2+^ [23,38].

Twelve replicates of each CIN concentration (0.2, 2, 20, 100, and 500 mg·L^−1^) were employed, with only water used for negative controls. The bulbs were positioned at the top of each 15 mL tube and incubated for 72 h at 25 °C, with the tested solutions refreshed every 24 h.

The EC_50_ and EC_10_ values—representing the effective concentrations of CIN causing 50% and 10% root growth, respectively, as well as their associated CI, were calculated based on the dose–response curves for *A. cepa* with the XLSTAT software (version 2025.1.3 (1431), Addinsoft, Lumivero, Paris, France 2025).

## 3. Results

The ecotoxicity of CIN was tested across four bioindicators. For the aquatic environment, the microcrustacean *D. magna* was used as a representative of freshwater ecosystems [39], alongside the bacterium *A. fischeri*, which allows for a sensitive and rapid assessment of toxicity in aquatic media [40]. Conversely, for the terrestrial environment, the earthworm *E. fetida*—a common indicator of soil health [41]—and the plant *A. cepa* were employed; the latter was usually selected to analyze genotoxic and phytotoxic effects within plant systems [42]. Furthermore, the study was complemented by a metabolic analysis of bacteria sourced from river water and soil to determine how this molecule impacts natural microbial communities. These communities perform key functions in organic matter degradation, nutrient cycling, and the stability of aquatic and terrestrial ecosystems [43,44,45,46]. By integrating all findings, this evaluation not only facilitates the study of the direct effects of the target compound on model organisms but also provides insight into the potential impact of its large-scale application on microbial communities and ecosystem dynamics.

### 3.1. Water Ecotoxicity

#### 3.1.1. Effect of *Trans*-cinnamaldehyde on Individual Organisms: *Aliivibrio fischeri* and *Daphnia magna*

The Gram-negative bacterium *A. fischeri* is widely recognized as a valuable indicator in ecotoxicity studies due to its bioluminescent capacity, a feature that renders the organism extremely sensitive to environmental stressors. Its bioluminescence, which is dependent on key metabolic processes, is readily altered by the presence of contaminants, enabling the precise and rapid detection of toxic substances in aquatic environments [40].

**Figure 1:**
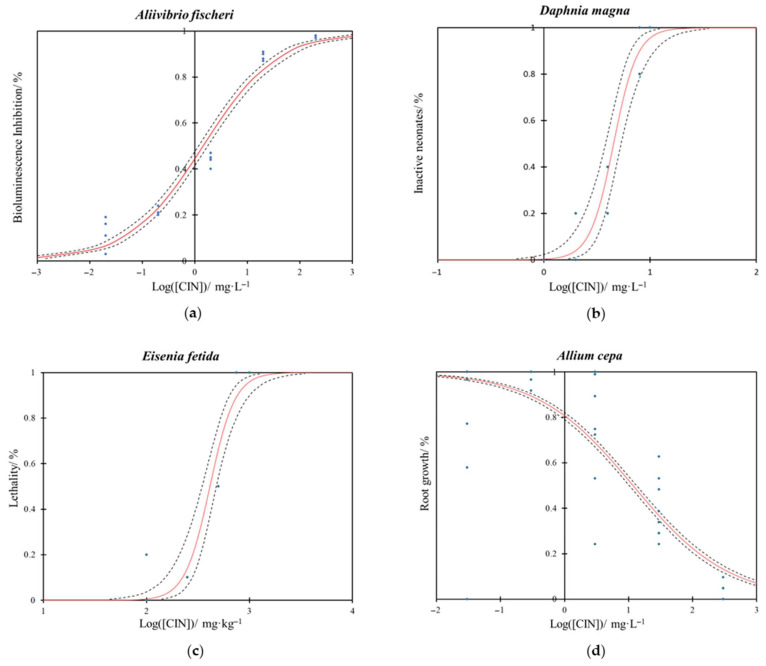
Dose–response curve for *trans*-cinnamaldehyde (CIN) in the (**a**) bioluminescence assay of *A. fischeri* after 30 min of exposure, (**b**) immobility assay of *D. magna* after 24 h of exposure, (**c**) lethality assay of *E. fetida* after 14 d of exposure and (**d**) root growth assay of *A. cepa* after 72 h of exposure. The curves represent the mean of four replicates. The red line indicates the model fit, the blue dots represent the experimental data, and the dashed black lines denote the 95% confidence interval limits.

The ecotoxicity study of CIN on *A. fischeri* demonstrates a clear dose–response relationship (Figure 1a) with high statistical significance of the model fitting (*p* < 0.0001). Effective concentrations 10% and 50% values, EC_10_ and EC_50_ respectively, for CIN were 0.039 mg·L^−1^ (95% Confidence Intervals (CI): 0.027–0.055 mg·L^−1^) and 1.428 mg·L^−1^ (CI: 1.172–1.741 mg·L^−1^), respectively (Table 1).

On the other hand, *D. magna* is a universally accepted model organism in aquatic ecotoxicology. Its high sensitivity to a wide range of chemicals, combined with its ecological relevance in aquatic food webs, positions it as an essential bioindicator for assessing the potential impacts of various substances on freshwater ecosystems. Furthermore, its standardized use in toxicity testing provides a robust basis for comparing data across different studies and chemical substances [5,25].

The dose–response curves obtained from the *D. magna* immobilization assay for CIN are shown in Figure 1b. Results indicate a high level of statistical significance of the model fitting (*p* < 0.0001). The EC_10_ and EC_50_ values for CIN were determined to be 2.581 mg·L^−1^ (95% CI: 1.787–3.179 mg·L^−1^) and 4.533 mg·L^−1^ (CI: 3.806–5.332 mg·L^−1^), respectively (Table 1).

#### 3.1.2. Effect of *Trans*-cinnamaldehyde on River Water Bacteria Communities: Average Well Color Development (AWCD) and Community Level Physiological Profile (CLPP)

The taxonomic characterization of the riverine water microbiota, conducted based on 16S rRNA gene sequences, was previously performed [23]. The obtained taxonomic profile was characteristic of a Mediterranean fluvial environment and consistent with previous research such as that of Pino-Otín et al. [47] and Battistuzzi & Hedges [48]. Briefly, this taxonomic analysis highlights a microbial community where primary producers, particularly diatoms (*Bacillariophyta*, 99.60% of the reads) of the genus *Thalassiosira*, are the most prevalent, accounting for 89.43% of this group. *Proteobacteria* represent the second largest phylum at 27.4%, with *Gammaproteobacteria* being the most frequent class within this group. Other significant phyla identified include Bacteroidetes and Actinobacteria, reflecting a high level of microbial diversity [23].

**Figure 2:**
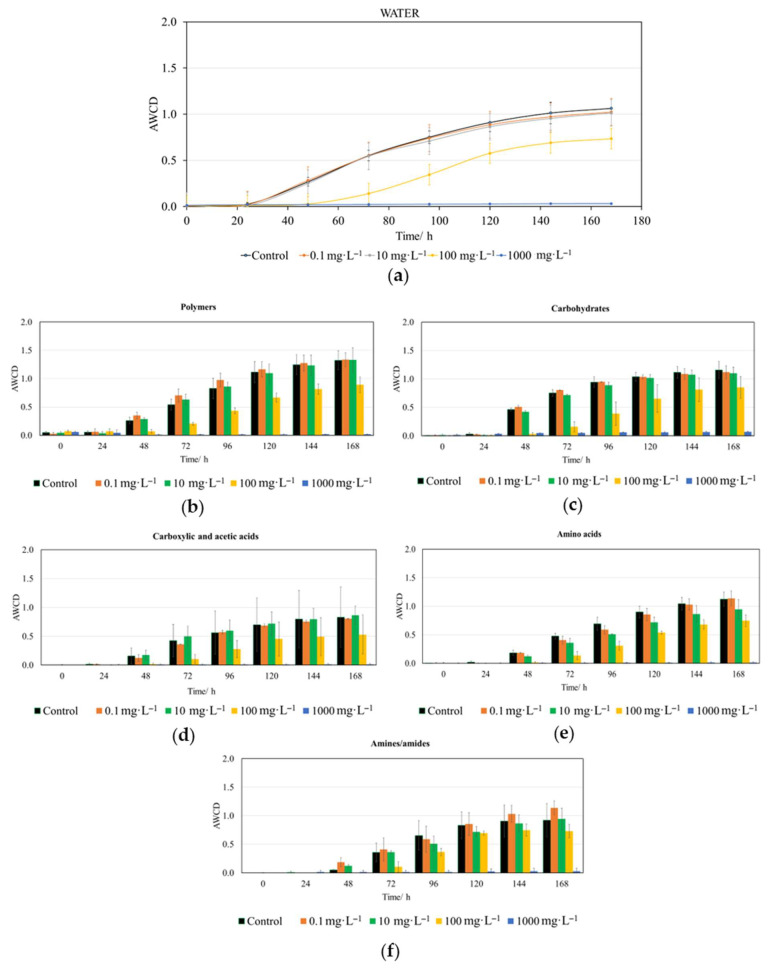
Impact of *trans*-cinnamaldehyde (CIN) on the metabolic activity of riverine aquatic microbiota. (**a**) Overall AWCD kinetics over time (h) for the whole plate. Substrate utilization profiles categorized by biochemical group: (**b**) polymers, (**c**) carbohydrates, (**d**) carboxylic and acetic acids, (**e**) amino acids and (**f**) amines and amides. Data are presented as the arithmetic mean of three replicates ± standard deviation (n = 3).

Changes in the metabolic profile of bacterial communities exposed to CIN were evaluated using the AWCD parameter over a 168-hour period at 24-hour intervals. These variations were analyzed for the whole plate (Figure 2a) as well as for distinct metabolite groups according to Weber & Legge [31], namely: polymers, carbohydrates, carboxylic and acetic acids, amino acids and amines/amides (Figure 2b–f). Statistically significant differences (*p* < 0.01) among the CIN concentrations for the previous experiments are offered in Table S1.

Our results (Figure 2 and Table S1) indicate that CIN exhibits a clearly dose-dependent toxicity toward aquatic microbiota. According to Table S1, significant differences in whole-plate AWCD only emerge after 48 h for the 100 and 1000 mg·L^−1^ concentrations compared to the control and lower doses (0.1 and 10 mg·L^−1^). This trend persists up to 72 h; however, from 96 h onward, significant differences are only observed between 1000 mg·L^−1^ and all lower concentrations, including the control.

Furthermore, the AWCD analysis by metabolite group—specifically, the CLPP—provides valuable information on how the tested compound, CIN, affects the ability of aquatic microorganisms to metabolize 31 of the most common organic carbon sources, distributed across the five groups previously mentioned (Figure 2b–f and Table S1). Regarding CIN, carbohydrate metabolism (Figure 2c) showed significant differences for concentrations of 100 and 1000 mg·L^−1^ compared to the control and lower concentrations starting from 48 h; however, from 120 h onward, only the 1000 mg·L^−1^ concentration exhibited significant differences relative to all others and the control (Table S1). The metabolism of polymers (Figure 2b) and carboxylic acids (Figure 2d) was affected in a very similar manner, showing significant differences between the 100 and 1000 mg·L^−1^ concentrations and both the lower doses and the control between 48 and 120 h (Table S1). After this point, only the 1000 mg·L^−1^ concentration significantly impacted the metabolism of these substrate groups. Regarding amino acid metabolism, a trend very similar to that of the two preceding groups could be observed. The most distinct behavior was presented by amines/amides (Figure 2f), in which statistically significant differences were only detected from 96 h onward and exclusively for the 1000 mg·L^−1^ concentration (Table S1).

The analysis of CLPP revealed that the metabolism of carbohydrates, polymers, carboxylic acids, and amino acids was the most sensitive to CIN exposure, especially during the first 120 h.

### 3.2. Soil Ecotoxicity

#### 3.2.1. Effect of *Trans*-cinnamaldehyde on Individual Organisms: *Eisenia fetida* and *Allium cepa*

The earthworm *E. fetida* plays a vital role in maintaining soil quality. The segmented body of the earthworm possesses a wall containing an epithelial layer rich in free nerve endings and specialized sensory cells [43]. These cells, concentrated in the first third of the body and on the ventrolateral and dorsolateral surfaces of each segment [49], act as chemoreceptors that detect substances such as sucrose and glycerol [50], as well as changes in salt concentration and environmental pH [51,52]. Chemosensation—defined as the ability to detect chemical irritants through the skin—is crucial for the earthworm’s survival. This sensory system not only enables the localization of food sources rich in microorganisms [53,54] but also facilitates social behaviors, such as group aggregation in response to adverse environmental conditions [52,55]. Due to its high sensitivity to chemical variations and the wide distribution of its cutaneous receptors, *E. fetida* is considered an ideal bioindicator for ecotoxicity studies.

In our study, the lethal toxicity of CIN for *E. fetida* was evaluated over 14 d through mortality tests, demonstrating that this compound has low toxicity regarding the survival of the species (Figure 1c). The fitting of the dose–response curve model showed a high statistical significance (*p* < 0.0001). LC_10_ and LC_50_ values were calculated at 223.151 mg·kg^−1^ (95% CI: 152.110–278.089 mg·kg^−1^) and 412.519 mg·kg^−1^ (95% CI: 346.666–476.449 mg·kg^−1^), respectively (Table 1).

On the other hand, the use of *A. cepa* as a bioindicator in environmental pollution assessment is extensively documented, given its high sensitivity to soil, air, and water conditions. As a globally significant horticultural crop, the onion has substantial nutritional requirements and exhibits marked sensitivity to soil pH variations [42,56]. These characteristics ensure that its development and yield are directly linked to soil quality, allowing this species to serve as a reliable indicator of the nutritional status of the edaphic environment. In this regard, concentrations of essential metabolites—such as nitrogen, sulfur, and potassium—have a substantial impact on the growth and quality of this crop, while its response to changes in soil acidity or alkalinity can reveal potential deficiencies or imbalances in soil composition [57].

In our study, following a 72 h exposure of *A. cepa* roots to CIN, a reduction in root elongation was observed, indicating that CIN exhibited certain toxicity toward the bulbs (Figure 1d). The model fitted the experimental results with high significance (*p* < 0.0001). Specifically, EC_10_ values for CIN were measured at 513.139 mg·L^−1^ (95% CI: 402.739–669.118 mg·L^−1^), while EC_50_ values for CIN corresponded to 11.644 mg·L^−1^ (95% CI: 10.231–13.276 mg·L^−1^), see Table 1.

#### 3.2.2. Effect of *Trans*-cinnamaldehyde on Soil Bacteria Communities: Average Well Color Development (AWCD) and Community Level Physiological Profile (CLPP)

The taxonomic profile of the soil microbiota, established through the analysis of the obtained 16S rRNA gene sequences, was determined previously by Ferrando et al. [23] and was consistent with the microbiota expected in this type of soil [25,48,58]. Succinctly, this taxonomic analysis reveals a complex soil microbial community dominated by the phyla *Proteobacteria* (45.06%) and *Actinobacteria* (35.35%). At more specific levels, the genera *Sphingomonas* and *Nocardioides* stand out for their high representativeness. The study [23] identified key functional groups, such as the class *Actinomycetales*, which are associated with organic matter decomposition, and *Sphingomonadales*, which account for a significant portion of the total reads. These results underscore the critical role these microbial populations play in essential ecological soil processes, including nutrient cycling and the degradation of organic compounds [23].

**Figure 3:**
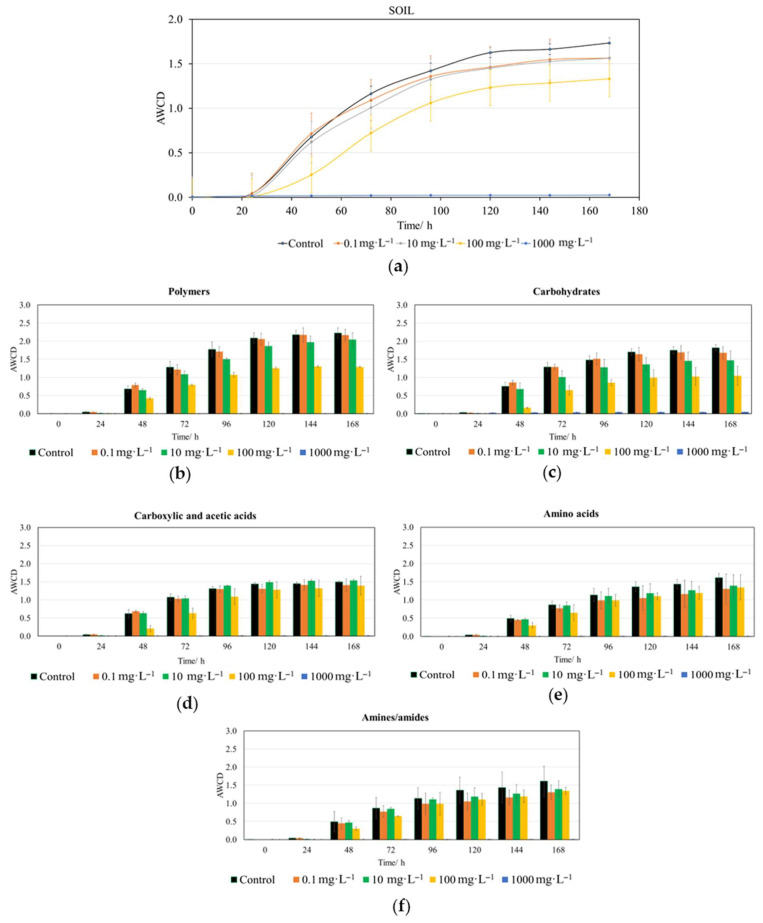
Impact of trans-cinnamaldehyde (CIN) on the metabolic activity of soil microbiota. (**a**) Overall AWCD kinetics over time (h) for the whole plate. Substrate utilization profiles categorized by biochemical group: (**b**) polymers, (**c**) carbohydrates, (**d**) carboxylic and acetic acids, (**e**) amino acids and (**f**) amines and amides. Data are presented as the arithmetic mean of three replicates ± standard deviation (n = 3).

Following a 168 h incubation in Biolog EcoPlates^TM^, AWCD values were evaluated for soil microbiota samples to subsequently analyze their capacity to metabolize different carbon sources when subjected to various concentrations of CIN. The results obtained in this study demonstrated that the toxicity of this compound manifested in a clearly dose-dependent manner in terrestrial microbial communities, mirroring the influence observed in aquatic systems (Figure 3 and Table S1).

The initial effects of CIN on the whole-plate AWCD were observed after the first 24 h (Figure 3a). From this point until 96 h, significant differences in absorbance values existed at concentrations of 100 and 1000 mg·L^−1^ compared to lower concentrations and the control (Table S1). It was from 120 h onward when significant differences only persisted for the 1000 mg·L^−1^ concentration when compared to all lower doses and the control.

In the corresponding CIN assays, the bacterial metabolism of polymers (Figure 3b) was affected from 24 h onward; starting at 48 h, significant differences began to appear for the 100 and 1000 mg·L^−1^ concentrations compared to lower doses and the control. These differences persisted until the end of the experiment (Table S1). Regarding carbohydrate metabolism (Figure 3c), the effect also began to manifest at 24 h, following a parallel behavior to that of polymers (Table S1). The same occurred for carboxylic and acetic acid metabolism (Figure 3d), but from 96 h onward only significant differences were observed for the 1000 mg·L^−1^ concentration with respect lower concentrations and the control (Table S1). At this same concentration, significant differences in the AWCD value (Table S1) for amino acids (Figure 3e) were identified from 48 h, and from 72 h in the case of amines/amides (Figure 3f).

A critical finding in this research is the differential impact of CIN on specific metabolic pathways. The metabolism of polymers and carbohydrates was affected early (24–48 h), whereas effects on carboxylic acids, amino acids, and amines appeared later and primarily at the highest dose (1000 mg·L^−1^).

## 4. Discussion

### 4.1. Water Toxicity

#### 4.1.1. Individual Organisms: *Aliivibrio fischeri* and *Daphnia magna*

To the best of our knowledge, ours is the first research to provide ecotoxicological data of CIN EC_10_ and EC_50_ in *A. fischeri*. These data (Table 1) were found to be in the range of EC_50_ values determined for other natural compounds, like eugenol (8.778 mg·L^−1^) [23], another major constituent of essential oils often compared with CIN. Nevertheless, the microbiological effects of CIN on the metabolism of *A. fischeri* were previously studied [59]. Brackman et al. [59] identified that CIN act as a Quorum Sensing (QS) inhibitor, a bacterial communication system crucial for regulating various functions, including bioluminescence. In *A. fischeri*, bioluminescence is partially controlled by the QS system, which depends on the accumulation of specific chemical signals to activate genes responsible for light production. CIN appears to interfere with this process by inhibiting QS signaling. This was demonstrated using synthetic fluorescent *E. coli* biosensors incorporating a modified version of the *A. fischeri lux* regulon [59,60]. Furthermore, Faleye et al. [61] studied the influence of CIN and several structural derivatives on various virulence factors in *Aliivibrio* spp. The compound demonstrated a remarkable capacity to interfere with multiple critical processes in *Aliivibrio parahaemolyticus*, inhibiting several phenotypes associated with virulence and colonization capacity in a dose-dependent manner. Among the affected properties, cell surface hydrophobicity, fimbriae production, and motility phenotypes—such as flagella-mediated swimming—are particularly noteworthy [61].

According to international classification criteria, such as the Globally Harmonized System [62], a substance with an EC_50_ (analyzed after 48 h of exposure) between 1 and 10 mg·L^−1^ when tested on *D. magna* is classified as Category 2, that is, “toxic to aquatic life”. Our result of 4.533 mg·L^−1^ (Table 1), although after an exposure of 24 h, falls precisely within this range. The literature data found for CIN EC_50_ in *D. magna* are scarce. The value of 3.86 mg·L^−1^ reported by the National Industrial Chemicals Notification and Assessment Scheme (NICNAS) [63], although measured after 48 h of exposure, is very similar to that experimentally obtained in our study. However, other authors, like Nwanade et al. [6], reported a 48 h LC_50_ of 28.34 mg·L^−1^.

As no more EC_50_ values for CIN on this specific microcrustacean were explicitly detailed in the literature, its toxicity can be contextualized by comparing it to other natural compounds. For instance, eugenol exhibits an EC_50_ of 1.963 mg·L^−1^ (95% CI: 1.571–2.444 mg·L^−1^) in *D. magna* [23]. This indicates that CIN is approximately as toxic as eugenol. Furthermore, when comparing *Cinnamomum zeylanicum* aqueous extracts to other aquatic models, its toxicity is much lower, as expected. For example, in brine shrimp (*Artemia salina*) assays, it demonstrated a LC_50_ of 38,980 mg·L^−1^ [64].

CIN is a highly reactive *α*, *β*-unsaturated aldehyde. Its toxicity in aquatic organisms like *D. magna* likely stems from its ability to induce oxidative stress through the production of reactive oxygen species (ROS) and the depletion of glutathione (GSH) [64]. This metabolic disruption could lead to energetic impairments, similar to the effects observed in *D. magna* exposed to other stressors that interfere with anaerobic metabolism and gluconeogenesis [65]. Additionally, the compound’s lipophilicity, characterized by a *n*-octanol–water partitioning coefficient (Log P) of 1.9, suggests a significant capacity for membrane interaction and penetration [66]. Furthermore, the filtering feeding behavior of *D. magna* may facilitate its uptake via the digestive route.

#### 4.1.2. River Water Bacteria Communities: Average Well Color Development (AWCD) and Community Level Physiological Profile (CLPP)

The dose-dependent toxicity behavior toward aquatic microbiota described previously is consistent with prior findings on the ecotoxicity of various compounds in river ecosystems, such as antibiotics and phenolic substances, where community growth—measured via AWCD—decreased as the concentration of the toxicant increased [25,47,67]. In our experiments, significant inhibition of the whole-plate AWCD was initially observed at 48 h for high concentrations (100 and 1000 mg·L^−1^), a temporal delay often associated with the adaptation phase of the community to chemical stress [47].

This aligns with observations made by Pino-Otín et al. [25], who reported that polymers, carbohydrates, and carboxylic acids were the functional groups most significantly impacted by high doses of antibiotics in river samples. Similarly, Valenzuela et al. [67] found that polymer metabolism was the primary metabolic function inhibited in river microorganisms exposed to the drug hydroquinone, reinforcing the idea that high-molecular-weight substrate degradation is particularly vulnerable to chemical stressors. Interestingly, the metabolism of amines in our study was only affected at 1000 mg·L^−1^ after 96 h, suggesting that the bacterial taxa responsible for utilizing nitrogenous compounds might possess intrinsic resistance or that these pathways are less susceptible to the specific mode of action of CIN at lower concentrations.

#### 4.1.3. *Trans*-Cinnamaldehyde Stability in Water

Regarding the environmental stability of the test compound, the toxicity observed in this study is considered representative of CIN exposure profile. In aquatic media, CIN reached complete degradation in natural well water over 30 days, although a 90% reduction occurred within the first 5 days [68]. The only detectable aquatic by-products, cinnamic acid and cinnamyl alcohol, dropped below significant thresholds after 10 days and possess lethality profiles similar to CIN in mammalian models [68]. Our experimental design aligns with these kinetics, as the aquatic assays (*A. fischeri* at 30 min and *D. magna* at 24 h) occurred while the parent compound was predominant. While previous lethality data relied on rat models, our targeted literature review for specific bioindicators found that the EC_50_ (15 min exposure) for *A. fischeri* was 11.41 mg·L^−1^ (95% CI: 1.7–75.45 mg·L^−1^) for cinnamic acid and 12.44 mg·L^−1^ (95% CI: 7.25–21.47 mg·L^−1^) for 4-hydroxycinnamic acid [69]. Additionally, the 24 h EC_50_ for 4-hydroxycinnamic acid on *D. magna* was determined to be 47.61 mg·L^−1^ (95% CI: 33.65–67.47 mg·L^−1^) [70]. Since these metabolite EC_50_ thresholds significantly exceed the values obtained in our study, the recorded ecotoxicity could not be justified by the accumulation of these sub-products and may be primarily ascribed to the action of CIN itself.

### 4.2. Soil Ecotoxicity

#### 4.2.1. Individual Organisms: *Eisenia fetida* and *Allium cepa*

Regarding the impact of CIN on *E. fetida*, no prior toxicity data are available; only Žabka et al. [71] tested an *Ocimum sanctum* extract with high cinnamate content and concluded that it exhibited no toxicity toward this organism. However, the range used did not exceed 300 mg·kg^−1^. The most studied pollutants in this bioindicator are chemical contaminants, especially heavy metals such as cadmium, lead, and mercury, which have shown adverse effects on its physiology, behavior, and reproduction, even at sublethal concentrations [72,73,74]. Nevertheless, research on the impact of natural compounds, such as essential oils and their main bioactive components, is much more limited. However, comparison with other soil-dwelling organisms and nematodes provides a relevant toxicological framework. For instance, CIN has demonstrated high efficacy against the plant-parasitic nematode *Meloidogyne incognita*, causing 97% mortality in second-stage juveniles (J2) at a concentration of only 52 mg·L^−1^ [75]. This concentration is significantly lower than the CIN EC_50_ observed for *E. fetida* in this study (Table 1), suggesting that its acute impact on larger soil macrofauna like earthworms may require higher exposure levels. Nevertheless, research on other nematodes such as *Caenorhabditis elegans* has shown that CIN acts as a strong anthelmintic agent, particularly when used in synergy with other natural compounds like condensed tannins [76].

CIN is known for its chemically distinct properties compared to other biopesticides. In nematodes, its activity has been linked to the inhibition of essential biological processes; specifically, related compounds like esters of cinnamic acid have been suggested to act through the inhibition of histone deacetylase [77]. In broader insect models such as *Tenebrio molitor*, CIN exposure resulted in significant morphological malformations and physiological disruptions [78]. For *E. fetida*, the mechanism likely involves systemic stress and disruption of metabolic pathways, similar to the effects observed in other non-target organisms where it can interfere with sensory responses or enzymatic activities [78].

As with *E. fetida*, there is a notable absence of prior studies concerning the phytotoxic effects of CIN on *A. cepa* root development. However, Alias et al. [79] determined that a CIN derivative produced slight toxicity in *A. cepa* roots at the maximum dose tested (100 mM, approx. 13.22 mg·L^−1^). Historically, cinnamon (*Cinnamomum* spp.) and its essential oils have been used as plant fertilizers and antifungal agents in traditional agricultural practices, noted for their ability to enhance plant growth and protect against pathogens. Depending on the part from which they are extracted, these oils have two main components, CIN and eugenol (1–80% and 5–95%, respectively), and are primarily effective in treating plant pathologies due to their antifungal, bactericidal, and insecticidal properties [80,81,82]. Although their effectiveness against these organisms is well-proven, none of these studies analyze the effect on the plant itself. Comparing results within the same assay parameters, Ferrando et al. [23] reported an EC_50_ of 23.116 mg·L^−1^ (CI: 20.061–26.743 mg·L^−1^) for eugenol. This suggests that the toxicological profile of this botanical structure is highly similar to CIN. On the other hand, Kowalska et al. [83] detected that treating seeds of various lettuce and parsley varieties with cinnamon oil at proportions exceeding 15% decreased field emergence and slowed growth, with signs of toxicity observed from that concentration onward, including drastic inhibitory effects on germination. Thus, we could postulate that the toxicity of the metabolite CIN, present in cinnamon, does occur and does so in a dose-dependent manner.

With respect to the mode of action of CIN on *A. cepa* no research was found. Only Dos Santos et al. [84] conducted a study to analyze the effect of cinnamon (not pure CIN). Results indicated that the components of this flavor additive caused cytotoxicity but not genotoxicity. Regarding these effects of flavorings on *A. cepa*, Souza Sales et al. [85] argued that the severe suppression of cellular division in healthy tissues may stem from agents that compromise the integrity of the mitotic spindle. This interference during mitosis subsequently led to substantial chromosomal disorganization.

#### 4.2.2. Soil Bacteria Communities: Average Well Color Development (AWCD) and Community Level Physiological Profile (CLPP)

To the best of our knowledge, no previous studies specifically analyzing the impact of CIN on soil microbial communities have been reported.

The results of this study demonstrate that CIN exerts a significant, dose-dependent inhibitory effect on the metabolic activity of soil microbial communities. The baseline taxonomic profile cited above, that is, Proteobacteria (45.06%) and Actinobacteria (35.35%), with *Sphingomonas* and *Nocardioides* as the most representative genera [23] are vital for soil ecological health, particularly in the degradation of organic compounds and nutrient cycling [23]. The observed reduction in AWCD of the whole plate suggests that only high concentrations of CIN (100 and 1000 mg·L^−1^) would significantly impair the functional diversity of these communities.

The immediate suppression of metabolic activity, observed after the first 24 h at high concentrations (Figure 3 and Table S1), aligns with the known antimicrobial properties of botanical volatile organic compounds [5,23]. For instance, studies on other biopesticides, such as the hydrolate of *Lavandula luisieri*, showed a similar capacity to decrease the growth and metabolic diversity of natural soil bacterial communities [86]. The fact that significant differences for the 1000 mg·L^−1^ dose persisted until the end of the 120 h incubation period indicates that high levels of CIN may cause a prolonged state of bacteriostasis or even localized microbial death, preventing the functional recovery of the community under the assay circumstances.

This selective inhibition would suggest that CIN may interfere with specific enzymes or membrane transport systems required for the breakdown of complex polymers. Similar metabolic shifts have been documented in studies evaluating the impact of phytogenic supplements and biopesticides on soil microbiota, where changes in carbon source utilization patterns often precede shifts in taxonomic composition [87].

Furthermore, the significant presence of *Actinomycetales* (20.81%) and *Sphingomonadales* (24.44%) [23] in the baseline soil suggests that the observed metabolic decline directly threatens key ecosystem services. *Sphingomonas* species are renowned for their ability to degrade recalcitrant organic matter and pesticides [86]; therefore, their inhibition by CIN could lead to a temporary reduction in the soil’s natural detoxification capacity.

Finally, the recovery of AWCD values at concentrations below 100 mg·L^−1^ after 120 h suggests a threshold effect. At lower doses, the soil microbiota may possess sufficient resilience—facilitated by the high initial representativeness of robust phyla like Actinobacteria—to metabolize or degrade the CIN, eventually restoring normal metabolic rates [87]. However, at 1000 mg·L^−1^, the chemical stress appears to overwhelm the community’s compensatory mechanisms.

#### 4.2.3. Trans-Cinnamaldehyde Stability in Soil

In terrestrial systems, CIN degrades more rapidly than in water; in fact, complete degradation occurs within 3 to 4 days across various soil types, with its six identified metabolites—including cinnamic acid and 4-hydroxycinnamic acid—undergoing total degradation within 3 days [1]. Our experimental design aligns with these kinetics, as the soil tests for *A. cepa* (72 h) occurred while the parent compound was predominant. Regarding the *E. fetida* bioassay, while the persistence and chemical integrity of CIN throughout the experimental period cannot be fully guaranteed, the EC_50_ and EC_10_ values (Table 1) accurately reflect the biological response under these specific experimental and standardized conditions. Unlike the other bioassays used in this research, which are shorter in duration, this study provides a more comprehensive insight into the impact on *E. fetida* following an initial exposure. Consequently, the observed results represent the toxicological effect of a single, non-sustained exposure event, regardless of the subsequent environmental fate of the compound within the soil matrix. In addition, for the 14-day *E. fetida* assay, the literature confirms that both CIN and its degradation by-products maintain a similar toxicological profile on rats [1]. Unfortunately, no bibliographic references for EC_50_ values in *E. fetida* or *A. cepa* were found for these metabolites, in contrast to the data available for CIN sub-products in aquatic media.

### 4.3. Potential Environmental Impacts of Trans-Cinnamaldehyde in Terrestrial and Aquatic Ecosystems

Despite its increasing use in cosmetics and biopesticides, no published data were found reporting environmental concentrations of CIN in rivers or soils. Given the dearth of relevant peer-reviewed literature, the findings of this study provide critical insights by demonstrating how CIN can impact non-target organisms.

Our tests on standard in vitro individual non-target aquatic organisms such as *D. magna* and *A. fischeri* revealed that CIN is toxic at 1–5 mg·L^−1^ after 24 h and 30 min exposures, respectively. Presence at these levels in aquatic bodies could cause significant population declines in primary consumers like *D. magna* or decomposer bacteria like *A. fischeri*. Such impacts would disrupt trophic webs, as microcrustaceans transfer energy from primary producers to higher levels and decomposers close carbon cycles.

However, several factors suggest that this acute in vitro ecotoxicity may not substantially affect real aquatic ecosystems.

First, CIN exhibits low environmental persistence, limiting acute lethal effects to localized, transient events rather than chronic threats. Degradation of CIN follows first-order kinetics, with degradation time (DT_50_) values of 1.58–13.14 d [68]. In freshwater or marine water, it degrades hydrolytically and microbiologically, with >70% eliminated within 10 d [63]. Second, in vitro data do not capture ecological realities, making community-level studies essential to reveal complex interactions. Our work with river-derived microbial communities showed that multi-species groups are more resistant than single-species ones, thanks to diverse coping mechanisms like toxicant degradation or enzymatic protection of neighbors. This resilience toward CIN likely stems from microorganisms such as *Pseudomonas* sp., identified in our samples [23,68]. This genus excels at degrading phenolic compounds like CIN [88]. Thus, while sensitive bacteria may decline, resistant ones fill the niche, preserving overall community growth and key functions such as nutrient cycling (C, N), pollutant degradation, primary production support, and water quality enhancement except at very high concentrations. Note that this applies to the parent compound; in situ metabolites could differ in activity, though evidence suggests no persistent toxicity [68].

A key finding is this microbial resilience: despite individual toxicity, community growth remained largely unaffected up to 10 mg·L^−1^. This aligns with Valenzuela et al. [67], who found river communities better tolerate toxins like hydroquinone than isolated *D. magna* or *A. fischeri*. Ferrando et al. [23] similarly highlighted protective diversity in multi-species systems, including collective degradation or sheltering of sensitive taxa when testing eugenol.

In terrestrial ecosystems, individual non-target organisms like *E. fetida* proved more resilient to CIN exposure. This earthworm is essential for soil quality, enhancing structure (porosity, aeration, water retention), accelerating organic matter decomposition, and enriching nutrients (N, P, organic C) through casts rich in enzymes and microbes. It also boosts microbial/fungal diversity, remediates contaminants (heavy metals, organics), supporting sustainable fertility. Therefore, its preservation is essential to maintain soil functionality [41].

Soil microbial communities similarly showed no effects below 10 mg·L^−1^, preserving core functions like nutrient cycling (C, N, P), aggregate formation, and remediation/plant health support [89].

The slight toxicity observed in *A. cepa* could be attributed to the fact that the standardized assay is conducted in water and in the absence of soil. As previously cited, CIN achieves over 70% biodegradability within 10 d and it might not produce toxic metabolites [77,90,91]. Quick leaching in diverse soils further minimizes prolonged exposure to non-target plants [91].

Compared to synthetic pesticides, such as those comprising the ‘Dirty Dozen’ (some of which are listed in Table 2) [92], CIN is a minimum of ten times less toxic to *D. magna* (the only bioindicator for which literature data were available). Furthermore, the environmental persistence of many of these synthetic molecules exceeds 56 to 96 days in water [93,94,95] and more than 12 months in soil [96]. When the ecotoxicity of CIN is contrasted with other biopesticides [97], such as rotenone or spinosad, the EC_50_ values for *D. magna* are at least ten times higher than those reported for these natural alternatives (Table 2). It should be noted that many biopesticides currently in use typically consist of complex mixtures of natural compounds rather than a single isolated active ingredient [97]; consequently, a direct comparison of their ecotoxicity with that of pure compounds may lack toxicological relevance. Regarding the environmental persistence of these biopesticides, it is pertinent to mention that there is still a significant lack of empirical literature on the subject.

Approved as a “basic substance” in the EU for organic farming, with lower persistent toxicity compared to synthetic alternatives, CIN can reduce cross-resistance risks compared to synthetics, aligns with sustainable initiatives like the EU Green Deal, and integrates effectively into Integrated Pest Management (IPM) strategies through controlled applications below these plant LC_50_ thresholds, safeguarding key soil invertebrates and microbiomes [90,91]. Thus, its use as an eco-pesticide represents a reduced environmental hazard compared to traditional synthetics and compared to other biopesticides.

## 5. Conclusions

Research to assess the ecotoxicity of CIN across different trophic levels was conducted. Our study evaluated this ecotoxicity on key non-target aquatic and soil organisms, including *A. fischeri*, *D. magna*, *A. cepa* and *E. fetida*, as well as the microbiota of their respective environments. To the best of our knowledge, this work provides the first acute toxicity data for CIN across these species, except for *D. magna*, where prior studies exist but used longer exposure times than our 24 h assays.

CIN exhibited acute toxicity toward primary aquatic consumers like *D. magna* and decomposers like *A. fischeri*, signaling potential risks to aquatic ecosystems at doses higher than 1 to 4 mg·L^−1^, respectively. However, this dose-dependent toxicity was markedly milder in water and soil microbiota, likely due to microbial resilience, rapid biodegradation, and differential susceptibility that favors tolerant strains. In fact, effects were observed at concentrations of CIN above 100 mg·L^−1^. Similar patterns emerged in soil microbial communities, where CIN showed very low toxicity to *E. fetida*, a critical invertebrate for soil quality, and only slight inhibition of *A. cepa* root elongation (EC_50_ of 11.644 mg·L^−1^). 

Nevertheless, the rapid environmental degradation of CIN, with 70% degradation in the first 10 days, in both terrestrial and aquatic systems offers a critical advantage by minimizing long-term toxicity effects. Its low persistence and reduced environmental risk in real aquatic ecosystems—coupled with minimal toxicity to key terrestrial communities—suggest that CIN’s use as a biopesticide poses no substantial ecological risks.

While the present study establishes the fundamental ecotoxicological profile of CIN under controlled conditions, further research involving commercial formulations, field-scale trials and persistence analysis, in both water and soil environments, of CIN alone and CIN as a part of commercial formulations will be essential to fully evaluate its behavior and safety under complex, real-world environmental dynamics.

## Figures and Tables

**Table 1:** Effective or Lethal concentration 10% (EC_10_ or LC_10_, respectively) and Effective or Lethal concentration 50% (EC_50_ or LC_50_, respectively), with the corresponding Confidence Intervals (CI) for *trans*-cinnamaldehyde (CIN) tests on *A. fischeri*, *D. magna*, *A. cepa* and *E. fetida*.

Non-Target Organisms	EC_10_ or LC_10_	CI	EC_50_ or LC_50_	CI
*Aliivibrio fischeri*	0.039 mg·L^−1^	(0.027–0.055) mg·L^−1^	1.428 mg·L^−1^	(1.172–1.741) mg·L^−1^
*Daphnia magna*	2.581 mg·L^−1^	(1.787–3.179) mg·L^−1^	4.533 mg·L^−1^	(3.806–5.332) mg·L^−1^
*Allium cepa*	513.139 mg·L^−1^	(402.739–669.118) mg·L^−1^	11.644 mg·L^−1^	(10.231–13.276) mg·L^−1^
*Eisenia fetida*	223.151 mg·kg^−1^	(152.110–278.089) mg·kg^−1^	412.519 mg·kg^−1^	(346.666–476.449) mg·kg^−1^

**Table 2:** Effective concentration 50% (EC_50_) for *D. magna* for some pesticides and biopesticides.

Pesticides	
	EC_50_
Compound	D. magna (mg·L^−1^)
Aldrin	0.0474 ^a^
Chlordane	0.0984 ^b^
DDT	0.005 ^c^ 0.0009–0.0019 ^d^ 0.350 ^e^
Endrin	2.4 ^f^ 0.059 ^g^
Heptachlor	0.078 ^h^
Hexachlorobenzene	>0.005 ^i^
Mirex	>0.034 ^j^
Toxaphene	>0.1 ^k^
Biopesticides	
Neem products	12.3–1128 ^l^
Rotenone	0.008 ^m^
Pyrethurm extract	0.0006–0.040 ^n^
Spinosad	0.48 ^o^

^a^ Ref [98]; ^b^ [99]; ^c^ [100]; ^d^ [101]; ^e^ [102]; ^f^ [103]; ^g^ [104]; ^h^ [105]; ^i^ [106]; ^j^ [107]; ^k^ [108]; ^l^ [109]; ^m^ [110]; ^n^ [111]; ^o^ [112].

## Data Availability

The original contributions presented in the study are included in the article/Supplementary Materials, further inquiries can be directed to the corresponding authors.

## Supplementary Materials

The following supporting information can be downloaded at: https://www.mdpi.com/article/10.3390/jox16030086/s1, Table S1: Statistically significant differences among the various *trans*-cinnamaldehyde (CIN) concentrations tested in the water and soil AWCD assays, both for the whole plate and for the different metabolite groups. Shaded cells indicate the doses at which significant differences were found (*p* < 0.01); Figure S1: Experimental procedure for AWCD and CLPP analysis of river water (blue) and soil (green) microbiota; Figure S2: Experimental procedure for *A. fischeri* (orange), *D. magna* (pink), *E. fetida* (grey) and *A. cepa* (black and white) toxicity test.

## Abbreviations

The following abbreviations are used in this manuscript:

AbsC	Optical Density of the control well lacking the carbon source
AbsW	Optical Density of each well containing the carbon source
AWCD	Average Well Color Development
CI	Confidence Interval
CIN	Trans-cinnamaldehyde or cinnamaldehyde
CLPP	Community Level Physiological Profile
DT	Degradation Time
EC	Effective Concentration
EPA	Environmental Protection Agency
EU	European Union
GSH	Glutathione
LC	Lethal Concentration
Log P	Logarithm of the n-octanol-water partitioning coefficient
NICNAS	National Industrial Chemicals Notification and Assessment Scheme
OD	Optical Density
QS	Quorum Sensing
ROS	Reactive Oxygen Species
USA	United States of America
