# Supplementary material for: Trans-Cinnamaldehyde as an Environmentally Low-Impact Phytosanitary: Evaluation of Its Toxicity Toward Aquatic and Terrestrial Non-Target Species
Source: J Xenobiot. 2026 May 16;16(3):86. doi: 10.3390/jox16030086 (PMC13214675; doi:10.3390/jox16030086)
Supplement: Supplementary file 1 [file jox-16-00086-s001.zip › jox-4247634-supplementary.pdf]

Table S1. Statistically significant differences among the various cinnamaldehyde (CIN) concentrations tested in the water and soil AWCD assays, both for the whole plate and for the different metabolite groups. Shaded cells indicate the doses at which significant differences were found ($p < 0.01$).

[CIN] mg·L^{-1}										
	WATER					SOIL				
	WHOLE PLATE									
**t=0 h**	C	0.1	10	100	1000	C	0.1	10	100	1000
C										
0.1										
10										
100										
1000										
**t=24 h**	C	0.1	10	100	1000	C	0.1	10	100	1000
C										
0.1										
10										
100										
1000										
**t=48 h**	C	0.1	10	100	1000	C	0.1	10	100	1000
C										
0.1										
10										
100										
1000										
**t=72 h**	C	0.1	10	100	1000	C	0.1	10	100	1000
C										
0.1										
10										
100										
1000										
**t=96 h**	C	0.1	10	100	1000	C	0.1	10	100	1000
C										
0.1										
10										
100										
1000										
**t=120 h**	C	0.1	10	100	1000	C	0.1	10	100	1000
C										
0.1										
10										
100										
1000										
**t=144 h**	C	0.1	10	100	1000	C	0.1	10	100	1000
C										
0.1										
10										
100										

1000										
**t=168 h**	C	0.1	10	100	1000	C	0.1	10	100	1000
C										
0.1										
10										
100										
1000										
**POLYMERS**										
**t=0 h**	C	0.1	10	100	1000	C	0.1	10	100	1000
C										
0.1										
10										
100										
1000										
**t=24 h**	C	0.1	10	100	1000	C	0.1	10	100	1000
C										
0.1										
10										
100										
1000										
**t=48 h**	C	0.1	10	100	1000	C	0.1	10	100	1000
C										
0.1										
10										
100										
1000										
**t=72 h**	C	0.1	10	100	1000	C	0.1	10	100	1000
C										
0.1										
10										
100										
1000										
**t=96 h**	C	0.1	10	100	1000	C	0.1	10	100	1000
C										
0.1										
10										
100										
1000										
**t=120 h**	C	0.1	10	100	1000	C	0.1	10	100	1000
C										
0.1										
10										
100										
1000										
**t=144 h**	C	0.1	10	100	1000	C	0.1	10	100	1000
C										
0.1										
10										
100										
1000										
**t=168 h**	C	0.1	10	100	1000	C	0.1	10	100	1000

C										
0.1										
10										
100										
1000										
**CARBOHYDRATES**										
**t=0 h**	C	0.1	10	100	1000	C	0.1	10	100	1000
C										
0.1										
10										
100										
1000										
**t=24 h**	C	0.1	10	100	1000	C	0.1	10	100	1000
C										
0.1										
10										
100										
1000										
**t=48 h**	C	0.1	10	100	1000	C	0.1	10	100	1000
C										
0.1										
10										
100										
1000										
**t=72 h**	C	0.1	10	100	1000	C	0.1	10	100	1000
C										
0.1										
10										
100										
1000										
**t=96 h**	C	0.1	10	100	1000	C	0.1	10	100	1000
C										
0.1										
10										
100										
1000										
**t=120 h**	C	0.1	10	100	1000	C	0.1	10	100	1000
C										
0.1										
10										
100										
1000										
**t=144 h**	C	0.1	10	100	1000	C	0.1	10	100	1000
C										
0.1										
10										
100										
1000										
**t=168 h**	C	0.1	10	100	1000	C	0.1	10	100	1000
C										
0.1										

10										
100										
1000										
**CARBOXYLIC AND ACETIC ACIDS**										
**t=0 h**	C	0.1	10	100	1000	C	0.1	10	100	1000
C										
0.1										
10										
100										
1000										
**t=24 h**	C	0.1	10	100	1000	C	0.1	10	100	1000
C										
0.1										
10										
100										
1000										
**t=48 h**	C	0.1	10	100	1000	C	0.1	10	100	1000
C										
0.1										
10										
100										
1000										
**t=72 h**	C	0.1	10	100	1000	C	0.1	10	100	1000
C										
0.1										
10										
100										
1000										
**t=96 h**	C	0.1	10	100	1000	C	0.1	10	100	1000
C										
0.1										
10										
100										
1000										
**t=120 h**	C	0.1	10	100	1000	C	0.1	10	100	1000
C										
0.1										
10										
100										
1000										
**t=144 h**	C	0.1	10	100	1000	C	0.1	10	100	1000
C										
0.1										
10										
100										
1000										
**t=168 h**	C	0.1	10	100	1000	C	0.1	10	100	1000
C										
0.1										
10										
100										

1000										
**AMINO ACIDS**										
**t=0 h**	C	0.1	10	100	1000	C	0.1	10	100	1000
C										
0.1										
10										
100										
1000										
**t=24 h**	C	0.1	10	100	1000	C	0.1	10	100	1000
C										
0.1										
10										
100										
1000										
**t=48 h**	C	0.1	10	100	1000	C	0.1	10	100	1000
C										
0.1										
10										
100										
1000										
**t=72 h**	C	0.1	10	100	1000	C	0.1	10	100	1000
C										
0.1										
10										
100										
1000										
**t=96 h**	C	0.1	10	100	1000	C	0.1	10	100	1000
C										
0.1										
10										
100										
1000										
**t=120 h**	C	0.1	10	100	1000	C	0.1	10	100	1000
C										
0.1										
10										
100										
1000										
**t=144 h**	C	0.1	10	100	1000	C	0.1	10	100	1000
C										
0.1										
10										
100										
1000										
**t=168 h**	C	0.1	10	100	1000	C	0.1	10	100	1000
C										
0.1										
10										
100										
1000										
**AMINES/AMIDES**										

**t=0 h**	C	0.1	10	100	1000	C	0.1	10	100	1000
C										
0.1										
10										
100										
1000										
**t=24 h**	C	0.1	10	100	1000	C	0.1	10	100	1000
C										
0.1										
10										
100										
1000										
**t=48 h**	C	0.1	10	100	1000	C	0.1	10	100	1000
C										
0.1										
10										
100										
1000										
**t=72 h**	C	0.1	10	100	1000	C	0.1	10	100	1000
C										
0.1										
10										
100										
1000										
**t=96 h**	C	0.1	10	100	1000	C	0.1	10	100	1000
C										
0.1										
10										
100										
1000										
**t=120 h**	C	0.1	10	100	1000	C	0.1	10	100	1000
C										
0.1										
10										
100										
1000										
**t=144 h**	C	0.1	10	100	1000	C	0.1	10	100	1000
C										
0.1										
10										
100										
1000										
**t=168 h**	C	0.1	10	100	1000	C	0.1	10	100	1000
C										
0.1										
10										
100										
1000										

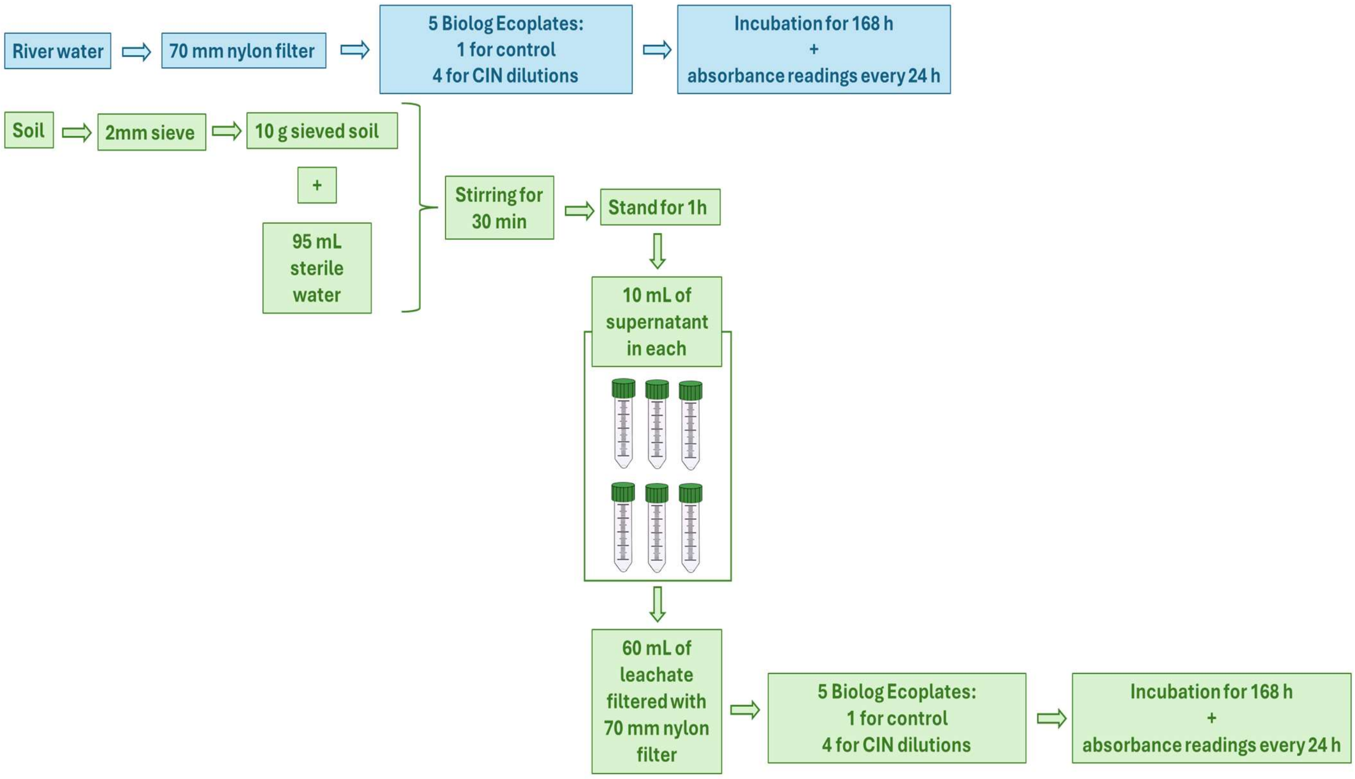

Figure S1. Experimental procedure for AWCD and CLPP analysis of river water (blue) and soil (green) microbiota.

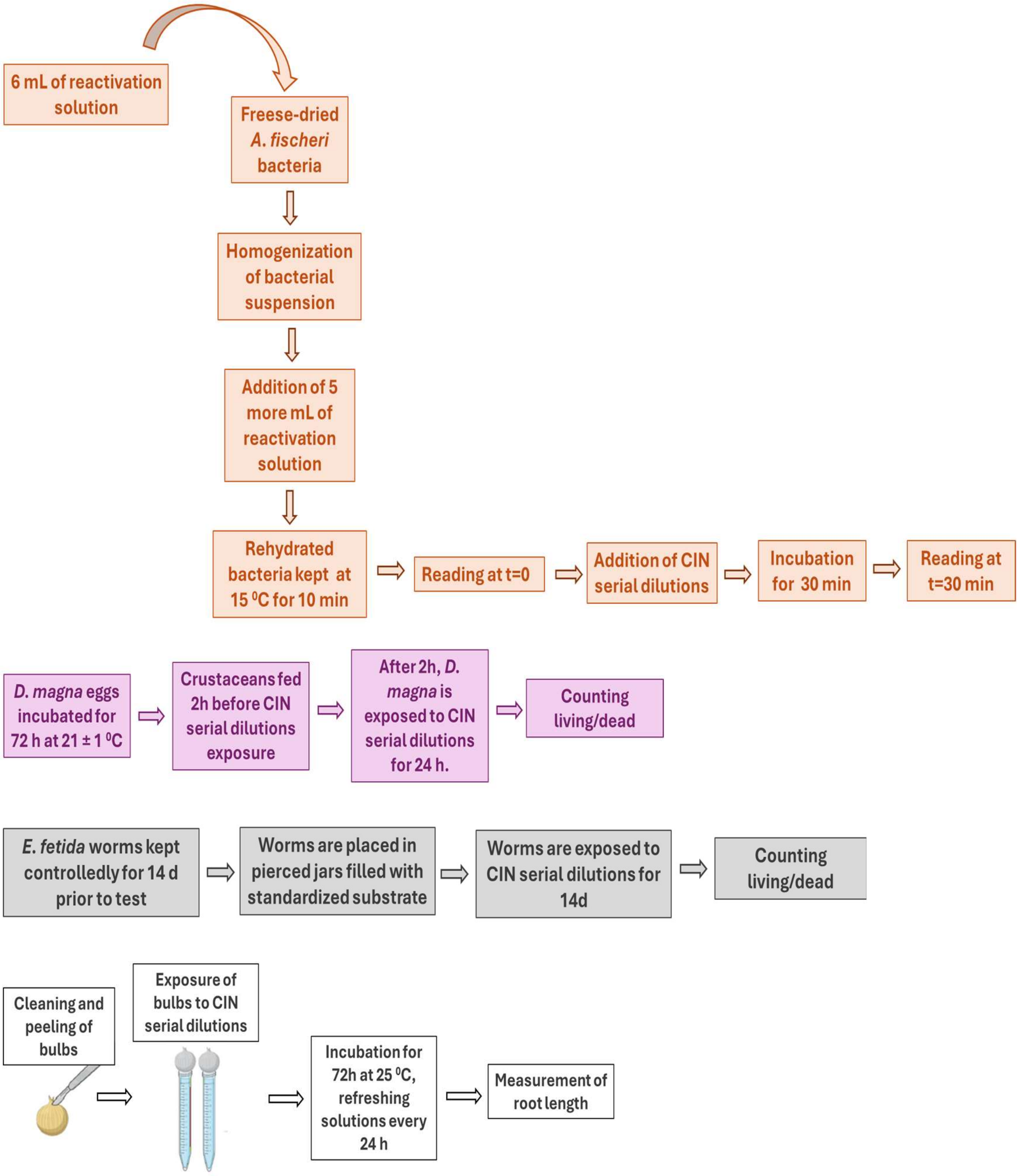

Figure S2. Experimental procedure for *A. fischeri* (orange), *D. magna* (pink), *E. fetida* (grey) and *A. cepa* (black and white) toxicity test.
